# Charitable Platforms in Global Surgery: A Systematic Review of their Effectiveness, Cost-Effectiveness, Sustainability, and Role Training

**DOI:** 10.1007/s00268-014-2516-0

**Published:** 2014-03-29

**Authors:** Mark G. Shrime, Ambereen Sleemi, Thulasiraj D. Ravilla

**Affiliations:** 1 Harvard Interfaculty Initiative in Health Policy, 14 Story Street, 4th Floor, Cambridge, MA 02138 USA; 2 Massachusetts Eye and Ear Infirmary, Boston, MA USA; 3 Department of Otology and Laryngology, Harvard Medical School, Cambridge, MA USA; 4 Program in Global Surgery and Social Change, Children’s Hospital, Boston, MA USA; 5 Maimonides Medical Center, New York, NY USA; 6 Mailman School of Public Health, Columbia University, New York, NY USA; 7 Lions Aravind Institute of Community Ophthalmology, Madurai, India

## Abstract

**Objective:**

This study was designed to propose a classification scheme for platforms of surgical delivery in low- and middle-income countries (LMICs) and to review the literature documenting their effectiveness, cost-effectiveness, sustainability, and role in training. Approximately 28 % of the global burden of disease is surgical. In LMICs, much of this burden is borne by a rapidly growing international charitable sector, in fragmented platforms ranging from short-term trips to specialized hospitals. Systematic reviews of these platforms, across regions and across disease conditions, have not been performed.

**Methods:**

A systematic review of MEDLINE and EMBASE databases was performed from 1960 to 2013. Inclusion and exclusion criteria were defined *a priori*. Bibliographies of retrieved studies were searched by hand. Of the 8,854 publications retrieved, 104 were included.

**Results:**

Surgery by international charitable organizations is delivered under two, specialized hospitals and temporary platforms. Among the latter, short-term surgical missions were the most common and appeared beneficial when no other option was available. Compared to other platforms, however, worse results and a lack of cost-effectiveness curtailed their role. Self-contained temporary platforms that did not rely on local infrastructure showed promise, based on very few studies. Specialized hospitals provided effective treatment and appeared sustainable; cost-effectiveness evidence was limited.

**Conclusions:**

Because the charitable sector delivers surgery in vastly divergent ways, systematic review of these platforms has been difficult. This paper provides a framework from which to study these platforms for surgery in LMICs. Given the available evidence, self-contained temporary platforms and specialized surgical centers appear to provide more effective and cost-effective care than short-term surgical mission trips, except when no other delivery platform exists.

## Introduction

Approximately 28 % of the global burden of disease is amenable to surgical intervention, a proportion that is higher in the developing world (author calculations, using the 2010 Global Burden of Disease survey [1]. Because of difficulties in access to care [2–5], at least part of this burden is borne by the international charitable sector. Historically, local hospitals in low- and middle-income countries (LMICs) have treated conditions associated with a low disability-adjusted life year (DALY) burden and have done so with a high loss to follow-up, especially as the complexity and upfront cost of surgeries increase [3]. Meanwhile, the charitable sector is large; in the United States, this sector, which includes many international surgical organizations, has grown at a pace exceeding GDP by 20 % [6]. This review will focus on the role of charitable organizations in surgical delivery in LMICs.

Any attempt to examine nongovernmental organizations (NGOs) must necessarily define these platforms. This is a daunting task—an entire galaxy of NGOs provides surgical care, few of which easily fit into any single categorization. Additionally, although the literature currently focuses on the conditions each organization treats, this focus masks salient similarities and differences among platforms, and, in doing so, may actually promote fragmentation in delivery.

This review, instead, will accomplish two goals: first, propose a classification scheme for charitable surgical delivery, focusing on the *method* of delivery, as opposed to the diseases treated. Using this new framework, this review will then compare NGO platforms along metrics of effectiveness, cost-effectiveness, sustainability, and their role in training. Focusing on the platform of care, rather than on disease-specific organizations, allows for benefits common to each platform to emerge, distinct from the diseases treated and the organizations that treat them.

We have limited our study only to charitable (or partly charitable) organizations and have evaluated them along only four domains: effectiveness, cost-effectiveness, sustainability, and their role in training. This is not to suggest that these are the only metrics by which these organizations should be evaluated. Ethical considerations are not, for example, explicitly considered, although they are arguably as important as the included domains [7–10].

Finally, other methods of delivering surgery in LMICs are not discussed: telemedicine [11] and cancer screening [12] are not included. Many individual surgeons organize their own trips to LMICs; none have produced peer-reviewed publications. Mobile surgical platforms sent from in-country hospitals [13], and surgical outreaches in humanitarian emergencies (as performed by organizations, such as Médecins Sans Frontières and the Red Cross) operate under different mandates, with currently limited (but positive) data, and are similarly excluded [14, 15]. Finally, teams that aim to establish residency or training programs have yet to publish enough of their outcomes to be evaluated. The few papers that have been published are, however, promising [16, 17].

## Methodology

A systematic review of the literature was performed to assess the cost, effectiveness, sustainability, and role in training of various surgical platforms. Guidelines and methods for systematic review have been standardized and reported elsewhere [18]. These guidelines, as they apply to observational studies, were followed in this paper. The MEDLINE search strategy is given in Box 1.

Bibliographies of the retrieved studies were searched for other relevant publications. Inclusion and exclusion criteria were decided on *a priori*. Only published, peer-reviewed articles were included. The search was not limited to articles in English. Data were extracted using piloted forms and performed by all three authors. Because of a high risk of heterogeneity in studies across multiple disease conditions, countries, and platforms of delivery, no mathematical summary measure was calculated.

**Fig. 1:**
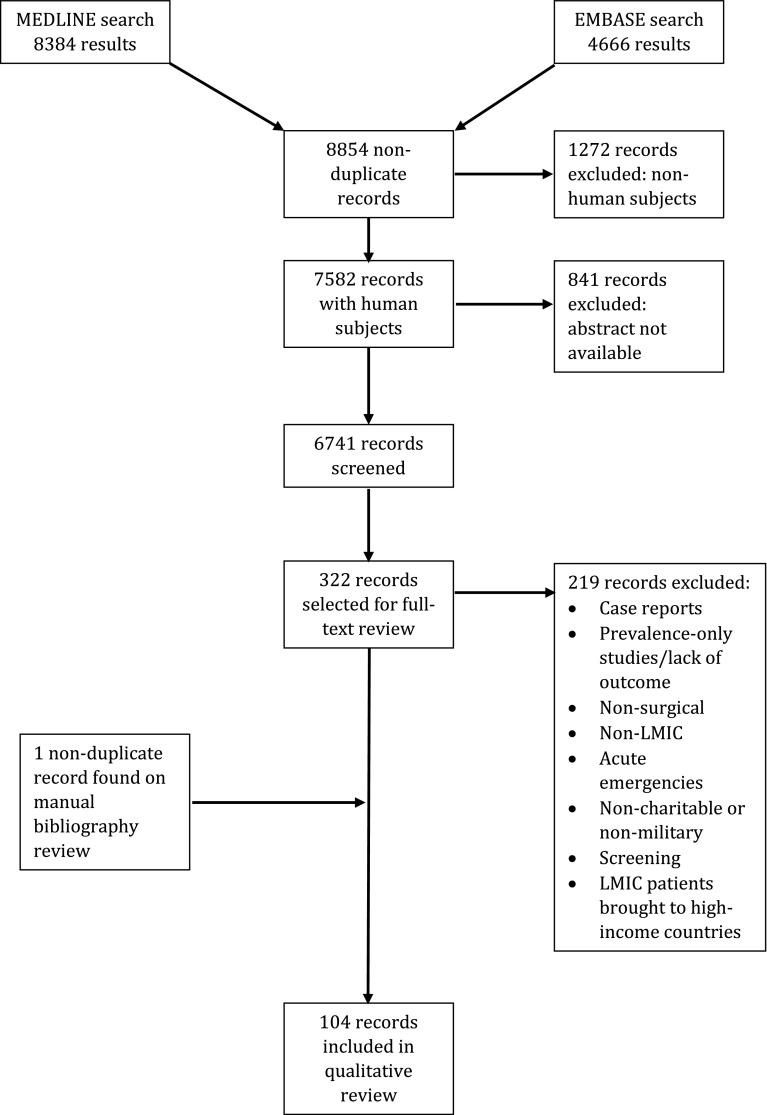
PRISMA diagram, documenting the search strategy results, inclusion criteria, exclusion criteria, and final records included in this qualitative systematic review

Of 8,854 records retrieved, 6,741 were screened by title and abstract; one additional article was found on bibliographic review, and the full text of 322 was screened. From these, 104 articles were selected inclusion. The review process, as well as the previously determined exclusion criteria are listed in the PRISMA diagram found in Fig. 1.

A note on terminology: although some NGOs providing surgery in LMICs are faith-based, not all are. The word *mission* in this review does not refer only to faith-based organizations; it is used more broadly of all temporary delivery platforms. Similarly, the word *humanitarian* is limited to missions that operate under the setting of acute emergencies, and the word *charitable* to organizations that are, at least in part, funded by private donations.

## Results

### A taxonomy of specialized surgical platforms

The literature suggests that charitable organizations delivery surgery in two basic ways: by establishing specialty surgical hospitals, or by focusing on more temporary platforms:

- *Temporary surgical platforms* By far the most common, this near-ubiquitous model of surgical delivery can be informatively broken down further:
  - *Short-term surgical trips* This platform sends surgeons, anesthesiologists, nurses, and/or supporting staff—along with, at times, surgical instrumentation and technology—into LMIC hospitals and clinics for short periods. Often, these NGOs perform a restricted set of surgeries, relying on local physicians for followup. Organizations such as Operation Smile [19–23], numerous orthopedic organizations [24], and many others fit this model.
  - *Self-contained surgical platforms* Significantly rarer, these NGOs often spend longer in-country than the short-term trips (months to years) but, importantly, carry their infrastructure with them. Self-contained on ships, airplanes, and other modes of transportation, these organizations tend not to leave behind any physical structure. Organizations such as Mercy Ships [25, 26] and CinterAndes fit this model.
- *Specialty surgical hospitals* Another common model for surgical delivery by NGOs, these platforms establish an entire physical plant, either *de novo* or within an existing structure, dedicated to the treatment of one or a few related surgical conditions. Organizations such as the Addis Ababa Fistula Hospital or the Aravind Eye Hospital fit this model.

This classification scheme allows conclusions to be drawn about effectiveness, cost-effectiveness, sustainability, and the role in training of broad platforms of charitable surgical delivery in LMICs, separate from the individual conditions treated.

### Temporary surgical platforms

#### Short-term surgical trips

Short-term, disease-specific surgical missions are myriad [27]: from “eye camps” in India [28–33] to “ear camps” in Namibia [34]; from organizations focused on facial clefting [19–23] to those focused on hernias [35], cardiac surgery [36], and endemic goiter [37]—services rendered, lengths of surgical trips, and resultant efficacies vary.

Underpinning these platforms, however, is a uniting model: surgical teams are flown into regions with high burdens of specific diseases, where they operate for short stints, often on the order of 1 to 2 weeks [38], and often in partnership with in-country physicians, to whom is left all but the most immediate of follow-up care. These missions, also called *safaris* [39] or *blitzes* [40], frequently carry their own equipment with them [38, 41], often return to the same region in subsequent years [24, 42–44], and strive toward close partnership with local hospitals and ministries of health [45, 46].

Despite the plethora of organizations that adopt this short-term model, evaluations of its effectiveness and cost-effectiveness are few. In part, this is due to a difficulty with follow-up. Of 4,100 operations for cleft lip and palate by 1 organization in 40 simultaneous sites, for example, only 703 patients (17 %) returned for a 6- to 9-month postoperative visit [19]. Similarly, in a Spanish-African cooperation program for the repair of hernias, follow-up was 21 % [16].

#### Effectiveness

A survey of 99 international surgical organizations found that the majority provided fewer than 500 operations per year [27]. Strong evidence exists for an association between surgical volume and outcomes in North America [47], with a stronger impact by *hospital* volume than by *surgeon* volume, especially for higher-complexity procedures [48, 49]. This seems to be maintained in the short-term platform; these organizations tend to suffer from higher mortality and complication rates while producing mixed results. In an evaluation of more than 17,000 operations performed in sub-Saharan Africa more than 114 surgical missions in two decades, an overall mortality of 3.3 % was achieved [50]. The majority of these operations were for hernias, for which a mortality as high as 1 % was observed—20 times higher than in high-income countries [51].

Both the success of an operative mission and its complication rates, however, vary by surgical procedure. Simpler procedures, such as tonsillectomy, appear safe when performed by short-term surgical missions [52]. Others less so: Maine et al [53]. Reported a rate oronasal fistula after cleft palate repair, which is more than 20-fold higher in surgical missions than in high-income countries. In their study, cases performed by experienced Ecuadorean and North American surgeons on a mission to Ecuador were compared with cases performed by similar surgeons at an American tertiary hospital. All surgeons showed this 20-fold increase in complication rates; no difference was found between Ecuadorean and North American surgeons. Although there are obviously patient-level factors that confound this increased complication rate, this finding lends further credence to an assertion that mission volume has potentially more impact than surgeon experience [53]. De Buys Roessingh et al. [42] similarly report relatively poor functional results in the repair of cleft palates on short-term surgical missions; the inherent difficulty of establishing a multidisciplinary approach in short-term surgical missions may contribute to these outcomes [54].

Results from cataract surgeries performed in eye camps are equally variable: Some report good vision outcomes [31], others poor [55]. Variability also is seen in otologic surgery; in surgical camps in Greenland [56, 57] and in mobile surgical units in Thailand [58], low complication rates and good results were found for chronic ear disease. Other authors, however, report success tied very strongly to either pathologic diagnosis [59] or the age of the surgical mission, with better results occurring a few years after the mission’s establishment [60].

Acceptable results have been found in cardiac surgery [36, 61], although some results come from very small surveys. Similar good results are reported in goiter missions, especially as they are repeated [37]. However, for the repair of burn contractures, Kim et al. found complications rates higher on surgical missions than in high-income countries [62], and, in orthopedics, Cousins et al. report success rates ranging from 28 to 75 %. Among the largest group of patients—those with lower limb trauma—47 % experienced complications [24]. Young et al. [63, 64] similarly document a not insignificant, postoperative infection rate after intramedullary nailing.

Overall, a pattern emerges in a review of the effectiveness of the short-term platform; for the condition most commonly treated by the charitable sector, the more complex the surgery, the more unsatisfactory the results. Both Marck et al [65]. and Huijing et al [66]. find this pattern, which combined with Maine’s findings above [53], leads them to recommend against short-term surgical missions for any but the simplest conditions [65, 66].

#### Cost-effectiveness

With a caveat to be discussed below, the few cost-effectiveness analyses that have been performed on surgical missions point to a beneficial cost-effectiveness ratio: cleft lip and palate repair costs anywhere from $52/DALY averted [67] to $1,827/DALY averted [23], or approximately $40 per patient [41], and benefit-cost analyses are similarly positive [68]. Orthopedic surgeries, at $340-$360/DALY averted, are slightly more expensive buys [38, 69].

These findings, however, must be interpreted with extreme caution, especially because they do not square with the assertion short-term surgical missions tend toward unsatisfactory outcomes. The apparent cost-effectiveness of surgical missions is an artifact of the way in which the analyses were conducted; almost all of the cited studies assume uncomplicated repairs, and all assumed that, without the mission, no surgery occurred. These assumptions will systematically result in a small cost-effectiveness ratio, biasing the analysis toward the charitable organization. As a result, an interpretation of these findings must be very narrow: only when *no other* platform treats the condition do these results imply that a surgical mission may be cost-effective. If the condition can be treated by other platforms—which, in many cases, it can—these cost-effectiveness results lose validity. This caveat should be combined with the fact that results of these cost-effectiveness studies depend on how the studies were conducted [70].

One cost-effectiveness analysis compared short-term platforms with other platforms for the treatment of one condition; Singh et al [55]. examined cataract surgeries performed at specialized eye camps, NGO hospitals, and the state medical college. Although not the worst value—that distinction fell to the state medical college—short-term eye camps were much less cost-effective than nongovernmental hospitals.

#### Sustainability and training

Many authors laud the salutary role that short-term surgical missions have in the education of surgical trainees in high-income countries [43, 71–85]. While this role is not to be dismissed, it cannot come at the cost of delivery of unsatisfactory care in LMICs [9, 86]. Besides one study, which documented an increase in laparoscopic surgeries after repeated training missions [17], no other evidence was found for the role of short-term missions in training.

Short-term surgical missions, however, have been put forward as a method to alleviate disease burden in LMICs. Unfortunately, the sustainability of this platform unclear. It is not altogether unlikely, for example, that these surgical camps treat the same conditions that are otherwise treated in local hospitals, and fragmentation in delivery contributes to an inability to meet the large burden of unmet need [87, 88]. The structure of the short-term medical mission itself may be detrimental to sustainability; patients are identified before the surgical team’s arrival, and the large volume of cases performed often disrupts local infrastructure, even after the team’s departure [40, 89].

Finally, although these platforms create awareness of surgery in the communities that they serve [90, 91], this awareness often can have counterintuitively *detrimental* effects on local infrastructure: when outcomes are consistently good, awareness influences positive health-seeking behavior in patients. Even the most sporadic bad outcomes, however, seem to discourage care-seeking outright [92].

Despite its ubiquity, the short-term surgical safari appears to have a relatively limited role in the delivery of surgical care. Given potentially unsatisfactory results, detrimental effects on health-seeking behavior, and stress on the local infrastructure, the short-term stand-alone surgical mission, when other options exist, is likely to be inefficient [93].

### Self-contained surgical platforms

The fact that complex procedures performed by short-term missions can yield unsatisfactory results [65, 66], combined with the fact that most local hospitals also are unable to provide this care consistently [3, 5, 94], leads to an obvious question. While LMICs improve their local infrastructure, how can the interim need be best met? Are specialized surgical hospitals (to be discussed next) the most effective and efficient method, or can a different temporary model, better structured than the short-term mission, provide effective surgical care?

Few examples of an intermediate model for surgical delivery exist, but those that do are promising. Mercy Ships, for example, maintains hospital ships, carrying an entire infrastructure (including pathology and radiology [26]), allowing them to provide ophthalmologic, reconstructive, general, orthopedic, and obstetric fistula surgeries [25, 95]. The few studies on the effectiveness of surgical procedures performed by this platform indicate outcomes comparable with those seen in high-income centers [25]. Military organizations adopt a similar model: the U.S. Navy maintains two hospital ships, which report mortality and complication rates that are equivalent to, if not better than, those found in high-income, country hospitals [96–98]. In addition, complex craniofacial surgeries, for which the short-term platform appears ill-suited, appear to be successfully performed by this platform [99]. There have been no cost-effectiveness evaluations of self-contained delivery platforms to date.

### Specialty surgical hospitals

#### Demand and supply constraints

Specialized surgical hospitals are myriad (see Box 2); many evolved from temporary surgical platforms. Cataract surgeries in India, for example, were initially performed in makeshift facilities before their care transferred to specialized hospitals. A population-based study, however, estimates that patients accessing short-term “eye camps” represent a mere 7 % of those in need [100], and current estimates put resource utilization of eye care facilities at 25 % [101].

Research by Browning and Patel, in the setting of obstetric fistula [93], similarly indicates that less than 1 % of surgical need for fistula repair is being met [93]. In Ethiopia alone, an estimated 9,000 women develop an obstetric fistula each year [102, 103]. Similar statements can be made about the unmet need for cardiac surgery, maternity services, and cancer care.

#### Effectiveness

Data for specialized surgical hospitals come primarily from ophthalmologic, fistula, and cancer centers [104, 105]. Although publications from specialized surgical hospitals treating other conditions exist, none include objective outcome measures [106, 107].

Evidence for the effectiveness and cost-effectiveness of specialty ophthalmologic hospitals has been presented above [55]; overall, they appear able to deliver high volumes of ophthalmologic surgery effectively [108]. A single publication from an eye hospital in Nigeria, however, reported poor postoperative vision outcomes [109]. Similarly, laparoscopic radical hysterectomy, other obstetric services, and repair of congenital anomalies can both be performed in LMIC specialized hospitals with outcomes similar to those found in the United States [105, 110–113].

Repair of obstetric fistulae is complex. Fistula surgeons are not considered expert until they have performed 300 cases, which may take years in short-term missions or local hospitals [114]. Even expert surgeons deliver closure and continence to only 85 % of patients. Both the Addis Ababa (a charitable organization) and Babbar Ruga (an initiative of the Nigerian government with some external funding) centers, however, report rates of successful fistula closure and return to continence of more than 90 % [115, 116].

Finally, complex surgical conditions, such as obstetric fistula and facial clefting, place specific design demands on the physical facility and require rehabilitative services [102]. While the local or district hospital may meet some of these needs, it must prioritize more life-threatening surgical conditions, making complex repair less likely [117]. In keeping with these findings, a recent expert elicitation study concluded that complicated obstetric fistulae are likely best repaired at high-volume, specialized surgical hospitals [118].

#### Cost-effectiveness

The single published, cross-platform comparison demonstrates the superior cost-effectiveness of permanent NGO hospitals in cataract surgery [55]. One other cost-effectiveness study published on surgery performed in the larger context of a mission hospital showed a beneficial cost-benefit ratio [119].

#### Sustainability and training

The Babbar Ruga fistula hospital reports having trained more than 600 fistula surgeons nurses worldwide [116]. Consistent with the above estimates [93], the experience of one author (AS) demonstrates the level of sustainability required for fistula training: the training of two Eritrean fistula surgeons required at least 5 years before competency levels and adequate case numbers were met. This is only possible in specialized platforms.

## Discussion

Surgical conditions constitute up to 28 % of the global burden of disease, and the current surgical infrastructure in many low-income countries cannot meet all of it. Access to surgical care is low [93, 101, 120], and most hospitals in LMICs do not treat high-DALY conditions [3]. Simultaneously, a rapidly growing, often fragmented charitable sector has stepped in to meet surgical need—a sector that has not been systematically evaluated [87].

Unfortunately, what evaluations have been done may actually promote fragmentation—examining surgical missions in isolation prevents informative similarities and differences from becoming explicit. We propose, instead, structuring evaluations around *platforms* for the delivery, not around disease types or individual missions. Doing so highlights the relative impact of models that underpin charitable surgery.

The overall findings from this systematic review are presented in Table 1. The literature suggests that NGOs deliver surgery by either establishing permanent surgical hospitals or in more temporary platforms—which themselves can be self-contained or can rely on local infrastructure.

**Table 1:** Summary of results (see text for further details)

Domain	Platform		
	Temporary, short-term	Temporary, self-contained	Surgical specialty hospital
*Effectiveness*	Poor results for complex procedures; effective for simple procedures	Potentially equivalent to developed-world outcomes	Equivalent to developed-world outcomes
*Cost-effectiveness*	Cost-effective if serving as the only platform for surgery; unlikely cost-effective otherwise	No data	Most cost-effective of the competing choices
*Sustainability*	Unlikely sustainable; may have a detrimental impact on health-seeking behaviour	No data	Platform suitable for sustainability
*Training*	Effective for training of developed-world surgeons. Little data on training of LMIC surgeons	Platform available for training	Definite role for training of LMIC surgeons

Sparse data on this platform limit the certainty of these conclusions

**Box 1:** MEDLINE search strategy

(Surgical Procedures, Operative[MeSH Terms] OR surgery[tiab] OR surgeries[tiab] OR surgical[tiab] OR operative[tiab] OR operating room[tiab] OR operation[tiab] OR cleft lip[tiab] OR cleft palate[tiab] OR eye[tiab] OR congenital[tiab] OR heart[tiab] OR cardiac[tiab] OR vesicovaginal[tiab] OR obstetric fistula[tiab] OR genital fistula[tiab] OR trauma[tiab])
AND
(Medical Missions, Official[MeSH Terms] OR Missions and Missionaries[MeSH Terms] OR Mobile Health Units[MeSH Terms] OR Relief Work[MeSH Terms] OR Voluntary Workers[MeSH Terms] OR humanitarian[tiab] OR surgical mission*[tiab] OR missionary[tiab] OR resource limited[tiab] OR low income countr*[tiab] OR middle income countr*[tiab] OR developing countr*[tiab] OR LMIC[tiab])
NOT “case reports”[publication type]

This search strategy (with appropriate language) also was used for EmBASE

**Box 2:** Examples of surgical specialty hospitals working in LMICs

Example surgical specialty hospitals working in low-resource settings
Cardiac
Salam Center, Khartoum, Sudan
Narayana Hrudayalaya Hospitals, Bangalore, India
Innova Children’s Heart Hospital, Hyderabad, India
Ophthalmic
ORBIS
Aravind Eye Hospitals, Tamilnadu, India
LRBT Eye Hospitals, Pakistan
Obstetric fistula
Babbar Ruga Hospital, Katsina, Nigeria
Hamlin Hospital, Addis Ababa, Ethiopia
Danja Fistula Center, Danja, Niger
Maternity services
Life Spring Hospitals, India
Cancer: Adayar Cancer Hospital, Chennai, India
Tata Memorial Hospital, Mumbai, India

The available evidence suggests that, despite its ubiquity, the short-term temporary surgical mission’s role should be limited to areas and conditions for which no other surgical delivery platform is available. In these settings, it delivers care efficiently. In settings in which alternative delivery systems exist, however, it appears much less effective [88]: short-term missions may not reach patients with unmet need [93]; risk delivering unsatisfactory results, especially around complex reconstructions [53, 65, 66]; often stress the local surgical infrastructure [40]; and may discourage health-seeking behavior [92], all of which undermine its sustainability.

In most cost-effectiveness analyses, short-term missions are compared against not providing any surgery and are assumed to be without complication [23, 38, 41, 67]. This overestimates their marginal effectiveness, systematically biasing analyses toward the surgical mission. In analyses in which the short-term platform is compared with other platforms, it becomes less cost-effective [55].

Self-contained temporary platforms are rare, but fit in the negative space between the short-term mission and the specialty hospital. They offer services usually not found in the short-term mission and are able to deliver care comparable to that found specialty hospitals in both LMICs and high-income countries [25, 26]. Cost-effectiveness studies have yet to be performed on this platform of delivery.

Finally, the literature suggests that specialized surgical centers might be effective in providing a high volume of care with good outcomes [115, 116]. Simultaneously, these permanent platforms are able to provide for some of the unique needs faced by patients with more complex conditions [102, 117, 121], and do so sustainably. One cost-effectiveness analysis demonstrates their increased efficiency over short-term camps [55], but further cost-effectiveness analyses are necessary.

This review is the first to attempt a broad, systematic evaluation of charitable surgical delivery in LMICs, distinct from the conditions treated and the individual organizations that treat them. As such, it has certain limitations. It should be noted, for example, that any taxonomy is leaky. Some organizations that establish hospitals *and* send short-term missions trips to other countries, some of the self-contained organizations have themselves established hospitals. That no classification system can adequately characterize any NGO does not, however, mean that research into these organizations must remain fragmented. This taxonomy, leaky though it may be, proposes a structure for future research into a large sector of the health system.

The peer-reviewed literature in this area is small, all outcomes studies are case series, and nearly all the cost-effectiveness are predicated on relatively heroic assumptions. In addition, although some studies do show less-than-optimal results, publication bias very likely exists. More importantly, a lack of evidence does not imply evidence of a lack. Many surgeons in LMICs, in addition to surgeons who work with these charitable organizations, have little time to devote to producing peer-reviewed publications. As such, a dearth of evidence exists as to the comparative effectiveness of NGO platforms and local hospitals within the same setting. This dearth highlights the need for further investigation into the effectiveness of surgery as delivered in these settings, as well as the potential role other research methods—such as realist synthesis—in the study of surgical delivery by charities in low- and middle-income countries.

Finally, of the domains along which delivery platforms were evaluated (cost-effectiveness, effectiveness, sustainability, and training), the former is controversial, especially given the various platforms used. Some organizations, for example, work entirely with volunteer staff; others pay. As such, these studies must be interpreted with caution.

## Conclusions

Despite these limitations, the classification scheme in this review allows for the first systematic evaluation of disparate charitable organizations. The charitable sector is large and spends a significant amount of donor money [6]. Limitations in the literature highlight the obvious need for more, and larger, evaluations of the effectiveness and cost-effectiveness of this sector’s role in the delivery of surgical care in LMICs. Determining the most effective platform for surgery stands to benefit patients, for whom this is often the only affordable avenue of care, while determining the most cost-effective platform stands also to align donor interests with those of the patients they seek to help. Finally, structuring future research around surgical delivery platforms will help in decreasing the fragmentation found in the nongovernmental world [3].
